# Preclinical disposition of MGS0274 besylate, a prodrug of a potent group II metabotropic glutamate receptor agonist MGS0008 for the treatment of schizophrenia

**DOI:** 10.1002/prp2.520

**Published:** 2019-09-13

**Authors:** Kohnosuke Kinoshita, Motoki Ochi, Katsuya Iwata, Misako Fukasawa, Jun‐ichi Yamaguchi

**Affiliations:** ^1^ Drug Metabolism and Pharmacokinetics Drug Safety and Pharmacokinetics Laboratories Research Headquarters Taisho Pharmaceutical Co., Ltd. Saitama Japan

**Keywords:** mGlu2/3 receptor, MGS0008, MGS0274 besylate, prodrug

## Abstract

MGS0274 besylate is an ester‐based lipophilic prodrug of a metabotropic glutamate (mGlu) 2 and mGlu3 receptor agonist MGS0008 and being developed for the treatment of schizophrenia. We investigated the disposition of these compounds in rats and monkeys and in vitro metabolism in humans to evaluate whether MGS0274 besylate could be useful as a prodrug in humans. After the oral administration of MGS0274 besylate to monkeys (2.89 mg/kg), MGS0008 was immediately found in plasma, reached a maximum concentration at 4 hours postdose, and decreased with a terminal half‐life of 16.7 hours; MGS0274 was barely detectable. The oral bioavailability as MGS0008 was 83.7%, which was approximately 20‐fold greater than that after oral dosing of MGS0008 (3.8%). In rats, MGS0008 penetrated the cerebrospinal fluid and was eliminated slower than from plasma. The in vitro metabolism study indicated that MGS0274 was rapidly hydrolyzed to MGS0008, which was not further metabolized. After the intravenous administration of MGS0008 to rats and monkeys, almost all the dose was excreted unchanged in urine. These results suggested that MGS0274 was, as expected, presystemically hydrolyzed to MGS0008 after gastrointestinal absorption and that MGS0008 was distributed throughout the body without further metabolism and ultimately excreted in urine in the animals. Furthermore, the hydrolytic activity against MGS0274 in the human liver S9 fraction was comparable to that in monkeys, suggesting the possibility of the rapid presystemic hydrolysis of MGS0274 to MGS0008 in humans, as it is in monkeys. Consequently, MGS0274 besylate is expected to function as a preferable prodrug in humans.

**Abbreviations**
AUC_0‐__∞_: area under the concentration‐time curve from time 0 to infinity
AUC_0‐t_: area under the concentration‐time curve from time 0 to t
CES: carboxylesterase
CL_renal_: renal clearance
CL_total_: total plasma clearance
C_max_: peak concentration
CNS: central nervous system
CSF: cerebrospinal fluid
CYP: cytochrome P450
GFR: glomerular filtration rate
HPLC: high‐performance liquid chromatography
LC‐MS: liquid chromatography‐mass spectrometry
LC‐MS/MS: liquid chromatography‐tandem mass spectrometry
mGlu: metabotropic glutamate
RI‐HPLC: high‐performance liquid chromatography equipped with radiochemical flow detection
RT‐PCR: real‐time polymerase chain reaction
SD: Sprague‐Dawley
t_1/2_: terminal half‐life
t_max_: time to peak concentration
Vd_ss_: volume of distribution at steady‐state

## 1 INTRODUCTION

**Figure 1:**
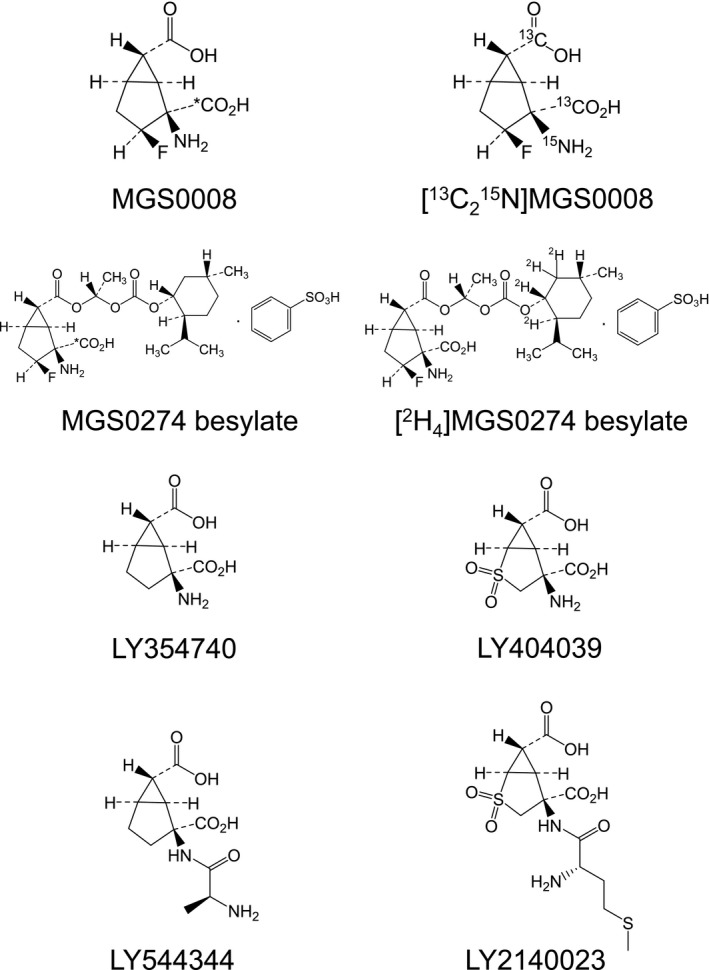
Chemical structures of the ^14^C or stable‐isotope labeled MGS0008 and MGS0274 besylate. Asterisk donates the position of the ^14^C label. For comparison, the chemical structures of LY354740, LY544344 (a prodrug of LY354740), LY404039, and LY2140023 (a prodrug of LY404039) are also shown^4^, ^31^

Group II mGlu (mGlu2/3) receptors play a role in regulating glutamatergic tone in the forebrain and limbic area, where glutamatergic dysregulation has been reported in patients with schizophrenia, and agonists for mGlu2/3 receptors are thought to be beneficial for the treatment of schizophrenia.^6^, ^28^, ^29^ LY2140023 discovered by Eli Lilly and Company, which is an orally bioavailable prodrug of the mGlu2/3 receptor agonist LY404039 (Figure 1), significantly improved positive and negative symptoms of schizophrenia in a phase 2 proof‐of‐concept trial, compared with placebo.^31^ However, LY2140023 failed to demonstrate efficacy in a phase 3 clinical trial.^1^ This raised questions as to whether mGlu2/3 receptors are actually therapeutic targets for schizophrenia. Thereafter, a pharmacogenetic analysis of the patients treated with LY2140023 showed that the single nucleotide polymorphisms located in the serotonin 2A receptor were associated with a change in the positive and negative syndrome scale, suggesting a pharmacogenetic relationship between the single nucleotide polymorphisms and the response to LY2140023 treatment.^21^ In addition, a *post hoc* analysis of data from phase 2 and phase 3 clinical trials for LY2140023 indicated that the patients with schizophrenia who had been ill for 3 years or less or previously treated with a dopamine D2 receptor antagonist exhibited the therapeutic response to LY2140023.^19^ Thus, the potential of mGlu2/3 receptors and their agonists as therapeutic targets and medications for schizophrenia is still worth investigating.

The mGlu2/3 receptor agonists, LY404039 and LY354740 (Figure 1), are rigid glutamate analogs and exhibited poor gastrointestinal absorption in humans (oral bioavailability of 3‐6%),^2^, ^8^ probably because of poor membrane permeability arising from their hydrophilic properties. Accordingly, a prodrug approach was inevitable to improve their oral bioavailability.^4^, ^31^

To improve the gastrointestinal absorption of hydrophilic compounds, transportable prodrugs or ester‐based lipophilic prodrugs are generally worth being developed.^32^ Both LY544344 (a prodrug of LY354740) and LY2140023 (Figure 1) were transportable prodrugs designed to be absorbed via intestinal peptide transporter 1 4, 30 and have indeed improved oral bioavailability in humans, compared with their parent compounds.^2^, ^18^ Although the transportable prodrug approach was successful, systemic exposure to the prodrug was still observed for LY2140023, accounting for roughly 30% of its parent compound in humans 31 and 15% of circulating radioactivity in monkeys.^2^ In general, higher exposures to pharmacologically inactive compounds, such as prodrugs themselves, should be avoided because these compounds can trigger toxicities and/or cause insufficient exposure to active components.^37^

MGS0008, (1*S*,2*S*,3*S*,5*R*,6*S*)‐2‐amino‐3‐fluorobicyclo[3.1.0]hexane‐2,6‐dicarboxylic acid (Figure 1), is a rigid glutamate analog with a potent agonist activity for mGlu2/3 receptors. In rat in vivo models known to be indicative of antipsychotic potential, treatment with MGS0008 significantly decreased phencyclidine‐induced locomotor hyperactivity and reduced conditioned avoidance responses, demonstrating that MGS0008 has antipsychotic potential.^27^, ^36^ Therefore, MGS0008 is expected to be useful for the treatment of psychiatric disorders, such as schizophrenia. However, based on the similarities in the chemical structures of MGS0008, LY354740, and LY404039, the possibility that MGS0008 might exhibit poor oral bioavailability in humans was easily predicted. Efforts to overcome this limitation were deemed vital for the future success of the clinical development of MGS0008. Accordingly, we decided to develop an ester‐based lipophilic prodrug to achieve not only good gastrointestinal absorption, but also the complete presystemic hydrolysis of the prodrug to MGS0008. On the basis of the results obtained from screening studies, such as chemical stability in buffers at pH1.2, 6.5, and 7.4, susceptibility to enzymatic hydrolysis in the human liver S9 fraction in comparison with marketed successful prodrugs as reference compounds, and oral bioavailability as the parent compound in animals, we discovered MGS0274 besylate, (1*S*,2*S*,3*S*,5*R*,6*S*)‐2‐amino‐3‐fluoro‐6‐({(1*S*)‐1‐[({[(1*R*,2*S*,5*R*)‐5‐methyl‐2‐(propan‐2‐yl)cyclohexyl]oxy}carbonyl)oxy]ethoxy}carbonyl)bicyclo[3.1.0]hexane‐2‐carboxylic acid monobenzenesulfonate (Figure 1).

In this study, we investigated the disposition of MGS0274 and MGS0008 in rats and monkeys, as well as their in vitro metabolism in humans to consider the developability of MGS0274 besylate as a prodrug. The results indicate that MGS0274 besylate is expected to function properly as a prodrug in humans.

## 2 MATERIALS AND METHODS

### 2.1 Materials

MGS0274 besylate was synthesized at Taisho Pharmaceutical Co., Ltd. (Saitama, Japan) by the method disclosed in the patent application.^12^ MGS0008, stable‐isotope labeled MGS0008 and MGS0274 besylate ([^13^C_2_^15^N]MGS0008 and [^2^H_4_]MGS0274 besylate), and MGS0039 ((1*R*,2*R*,3*R*,5*R*,6*R*)‐2‐amino‐3‐[(3,4‐dichlorophenyl)methoxy]‐6‐fluorobicyclo[3.1.0]hexane‐2,6‐dicarboxylic acid) were also synthesized at Taisho Pharmaceutical Co., Ltd. The ^14^C radiolabeled compounds of MGS0008 (specific activity: 2.07 GBq/mmol) and MGS0274 besylate (specific activity: 2.07 GBq/mmol), of which the radiochemical purities were > 99%, were synthesized by Quotient Bioresearch (currently Pharmaron, Wales, UK). The chemical structures of the ^14^C or stable‐isotope labeled compounds of MGS0008 and MGS0274 besylate are shown in Figure 1. All other reagents were of analytical grade or of high‐performance liquid chromatography grade. Plasma (anti‐coagulant: EDTA‐2K) and serum from Sprague‐Dawley (SD) rats were obtained from HAMRI (Ibaraki, Japan) and Charles River Laboratories Japan (Kanagawa, Japan), respectively. Plasma (anti‐coagulant: EDTA‐2K) and serum from cynomolgus monkeys were purchased from HAMRI. Plasma (anti‐coagulant: sodium heparin) and serum from humans were obtained from KAC (Kyoto, Japan) and Kohjin Bio (Saitama, Japan), respectively. Cryopreserved hepatocytes prepared from rats and monkeys were purchased from Xenotech (Kansas City, KS), and cryopreserved human hepatocytes were obtained from BioreclamationIVT (currently BioIVT, Westbury, NY). Rat and monkey tissue S9 fractions of the intestine, liver, lung, and kidney were obtained from Xenotech. Human tissue S9 fractions were purchased from BioreclamationIVT (intestine) and Xenotech (liver, lung, and kidney).

### 2.2 Animals

Male Wistar rats (7 weeks old) were purchased from Charles River Laboratories Japan. The animals were maintained under controlled temperature (23.3 ± 3°C) and humidity (50 ± 20%) conditions with a 12‐hour light/dark cycle at Taisho Pharmaceutical Co., Ltd. Food and water were provided ad libitum except during the tests. Experiments involving male cynomolgus monkeys (6‐8 years old; Beijing Grandforest Trading, Beijing, China) were conducted at HAMRI, and the animals were maintained under controlled temperature (24 ± 3°C) and humidity (50 ± 20%) conditions with a 12‐hour light/dark cycle. Food and water were provided ad libitum. All the animal experimental procedures involving animal handling were approved by the Institutional Animal Care and Use Committee of Taisho Pharmaceutical Co., Ltd. (rats and monkeys) and/or HAMRI (monkeys).

### 2.3 Plasma protein binding

[^14^C]MGS0008 was spiked with rat, monkey, or human plasma at final concentrations of 0.1, 1, and 10 μg/mL. The protein binding was evaluated according to a previously published method.^11^ MGS0274 besylate was spiked with monkey or human plasma at final concentrations of 0.1, 1, and 10 μg/mL as MGS0274, which was placed in a donor compartment of a 96‐well equilibrium dialysis plate (Dialysis Membrane Strips MWCO 12‐14K; HT Dialysis, Gales Ferry, CT). Sodium phosphate buffer (0.05 M, pH7.4) containing sodium chloride (0.07 M) was placed in a reservoir compartment. After incubation for 5 hours at a rate of 100 rpm at 37°C in humidified air‐5% CO_2_, aliquots of the plasma and dialysate were collected from each compartment, mixed with acetonitrile/methanol (9:1, v/v) containing [^2^H_4_]MGS0274 besylate, and centrifuged. Aliquots of the supernatant were subjected to bioanalysis using liquid chromatography‐tandem mass spectrometry (LC‐MS/MS) (see Table S1‐A). The protein binding (%) was calculated based on the instruction manual of the 96‐well plate.

### 2.4 In vitro metabolite profiling of [^14^C]MGS0274 in rats, monkeys, and Humans

Rat, monkey, or human cryopreserved hepatocytes in Leivovitz L‐15 medium at 0.5 million cells/mL were incubated at 37°C for 1 hour with [^14^C]MGS0274 besylate at 10 μmol/L. The reaction was terminated by adding 10% w/v trichloroacetic acid solution, and the resultant mixture was centrifuged. Aliquots of the supernatant were subjected to bioanalysis using high‐performance liquid chromatography equipped with radiochemical flow detection (RI‐HPLC) (see Table S1‐*B*). Aliquots of the supernatant from unlabeled incubation samples and authentic standards were also injected into a liquid chromatography‐mass spectrometry (LC‐MS) system under the same chromatographic conditions to compare their chromatographic retention times and mass spectral data (see Table S1‐*B*).

### 2.5 Hydrolytic activity against MGS0274 in rat, monkey, and human sera and tissue S9 fractions

Rat, monkey, or human serum that had been pre‐incubated for 5 minutes at 37°C was spiked with MGS0274 besylate at 10 μmol/L. After incubation for 5 to 60 minutes, the reaction was terminated by adding acetonitrile/methanol/formic acid (90:10:1, v/v/v) containing [^13^C_2_^15^N]MGS0008, and the resultant mixture was centrifuged. Tissue S9 fractions with final protein concentrations of 0.25 mg/mL were pre‐incubated for 5 minutes at 37°C, then spiked with MGS0274 besylate at 10 μmol/L. After incubation for 1 to 5 minutes, the reaction was terminated by adding acetonitrile/methanol/formic acid (90:10:1, v/v/v) containing [^13^C_2_^15^N]MGS0008, and the resultant mixture was centrifuged. Aliquots of the supernatant were subjected to bioanalysis using LC‐MS/MS (see Table S1‐*C*). The formation rate of MGS0008 from MGS0274 in the reaction mixture was calculated using the following equation:

$$\text{Formation rate}\left(\text{nmol}/\min/\text{mg protein}\right)=\left(\text{concentration of MGS0008},\text{nmol/mL}\right)/\left(\text{incubation time},\min\right)/\left(\text{protein concentration},\;\text{mg protein}/\text{mL}\right)$$

### 2.6 Inhibition of cytochrome P450s

The inhibition potential of MGS0008 (0.3‐100 µmol/L) and MGS0274 besylate (0.03‐10 µmol/L) on the activity of human cytochrome P450 (CYP) isoforms (CYP1A2, 2B6, 2C8, 2C9, 2C19, 2D6, and 3A) was investigated using probe substrates for each CYP isoform and human liver microsomes, of which the final protein concentration was set at 0.1 mg/mL (see Table S2). The assay was started by spiking the reaction mixture, which had been pre‐incubated at 37°C for 5 minutes, with NADP^+^. After incubation for 5 to 10 minutes, the reaction was terminated by adding acetonitrile containing an internal standard for each probe substrate, and the resultant mixture was centrifuged. Aliquots of the supernatant were subjected to bioanalysis using LC‐MS/MS (see Table S1‐D).

### 2.7 Induction of cytochrome P450s

The primary cultured cryopreserved human hepatocytes were plated in a collagen‐coated 96‐well plate (Becton Dickinson, Franklin Lakes, NJ) with Williams’ Medium E (Invitrogen, Carlsbad, CA) containing Geltrex^TM^ (Invitrogen) and Maintenance Supplements (Invitrogen) and maintained in humidified air‐5% CO_2_ at 37°C for 3 days. Then, the cells were cultured for three consecutive 24‐hour treatments with MGS0008 (3‐30 µmol/L), with MGS0274 besylate (3‐30 µmol/L), or with positive controls (omeprazole for CYP1A2 at 50 µmol/L, phenobarbital for CYP2B6 at 1000 µmol/L, or rifampicin for CYP3A4 at 10 µmol/L). The mRNA expression levels of CYP1A2, 2B6, and 3A4 were measured using the real‐time polymerase chain reaction (RT‐PCR) method (7500 Fast RT‐PCR system; Applied Biosystems, Foster City, CA) with TaqMan^®^ Fast Universal PCR Master Mix (probe ID: Hs00167927_m1 for CYP1A2, Hs04183483_g1 for CYP2B6, and Hs00604506_ml for CYP3A4; Applied Biosystems). The fold change in the mRNA expression was calculated using the 2^‐ΔΔCT^ method.^22^

### 2.8 Pharmacokinetics in rats and monkeys

The pharmacokinetic profiles of MGS0008 and MGS0274 were investigated in fasted male Wistar rats (n = 3) and fed male cynomolgus monkeys (n = 4). Monkeys had been given 60 g of a primate diet (PS‐A; Oriental Yeast, Tokyo, Japan) one hour before administration. For intravenous administration, MGS0008 was dissolved in saline (pH7.4) and administered to rats (3 mg/kg) and monkeys (1 mg/kg). Blood samples were collected from the tail vein (rats) or the forelimb cephalic vein (monkeys) into tubes containing EDTA‐2K at 5, 15, and 30 minutes and at 1, 2, 4, 8, and 24 hours postdose for both species, and an additional sample was collected at 12 hours postdose for monkeys. Urine samples were collected until 48 hours at 24‐hour intervals (rats) or at 0‐ to 8‐hour, 8‐ to 24‐hour, and 24‐ to 48‐hour intervals (monkeys). The plasma or urine samples was mixed with acetonitrile/methanol (9:1, v/v) containing [^13^C_2_^15^N]MGS0008 and centrifuged. Aliquots of the supernatant were subjected to bioanalysis using LC‐MS/MS (see Table S1‐E). For oral administration, MGS0008 was dissolved in distilled water (rats) or 0.5% w/v methylcellulose 400 (monkeys) and administered to rats (3 mg/kg) and monkeys (1 mg/kg). MGS0274 besylate was suspended in 0.5% w/v methylcellulose 400 containing 0.1% v/v Tween 80 and administered to monkeys at a dose of 2.89 mg/kg (1‐mg equivalent of MGS0008/kg). Blood samples were collected from the tail vein (rats) or the forelimb cephalic vein (monkeys) into tubes containing EDTA‐2K at 30 minutes, 1, 1.5, 2, 4, 6, 8, and 24 hours postdose for rats, and 30 minutes, 1, 2, 4, 8, 12, and 24 hours postdose for monkeys. The plasma samples were mixed with acetonitrile/methanol (9:1, v/v) containing internal standards ([^13^C_2_^15^N]MGS0008 and MGS0039) and centrifuged. Aliquots of the supernatant were subjected to bioanalysis using LC‐MS/MS (see Tables S1‐E and F). In the study examining the tissue distribution of MGS0008 in rats (3 mg/kg), blood samples were collected from the posterior vena cava into tubes containing EDTA‐2K under isoflurane anesthesia at 1.5, 4, 6, or 24 hours postdose. Then, the animals were euthanized by exsanguination from the abdominal aorta; cerebrospinal fluid (CSF) samples were drawn from the spinal cord space in each animal. The plasma or CSF was mixed with acetonitrile/methanol (9:1, v/v) containing [^13^C_2_^15^N]MGS0008 and centrifuged. Aliquots of the supernatant were subjected to bioanalysis using LC‐MS/MS (see Table S1‐*E*).

### 2.9 Pharmacokinetic analysis

The plasma concentration‐time profiles of MGS0008 and MGS0274 were analyzed using a non‐compartmental analysis method with pharmacokinetic analysis software Phoenix^®^ WinNonlin^®^ 6.2 or later (Certara, Princeton, NJ).

### 2.10 Prediction of human pharmacokinetic parameters

The prediction of CL_total_, Vd_ss_, and t_1/2_ in humans was performed using single‐species allometric scaling methods 14 with the pharmacokinetic parameters obtained from the intravenous administration of MGS0008 to rats and monkeys, unbound fraction of MGS0008 in rat, monkey, and human plasma (1.0), and body weight of rats (0.3 kg), monkeys (5 kg), and humans (60 kg).

## 3 RESULTS

### 3.1 Plasma protein binding

The unbound fractions of MGS0008 in rat, monkey, and human plasma were found to be 103.5‐106.2%, 103.8‐105.1%, and 106.0‐109.6%, respectively, indicating that MGS0008 hardly binds to plasma proteins in all the species tested. Regarding MGS0274, the protein binding levels in monkey and human plasma were found to be 92.4‐93.5% and 95.7‐96.1%, respectively. The protein binding of MGS0274 in rat plasma was not tested because of its rapid hydrolysis to MGS0008.

### 3.2 In vitro metabolite profiling of MGS0274 in rats, monkeys, and humans

**Figure 2:**
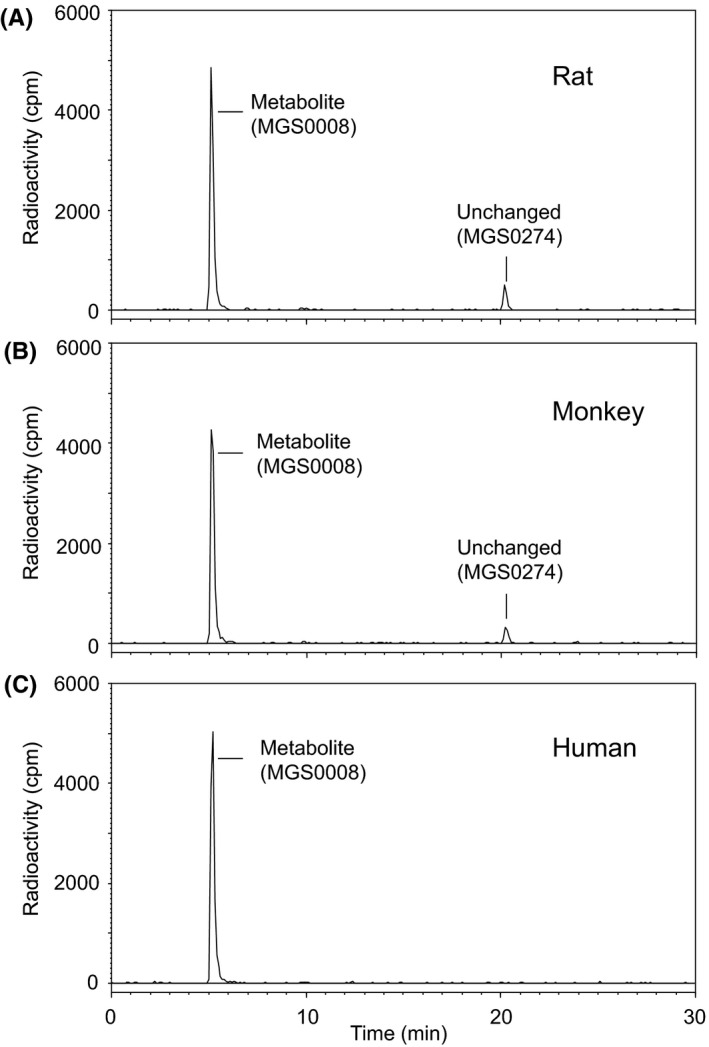
HPLC‐radiochromatograms of the incubation mixtures of [^14^C]MGS0274 besylate with (A) rat, (B) monkey, and (C) human cryopreserved hepatocytes. Rat, monkey, or human cryopreserved hepatocytes at 0.5 million cells/mL were incubated at 37°C for 1 h with [^14^C]MGS0274 besylate at 10 μmol/L. In all the species tested, MGS0274 was nearly and completely hydrolyzed to MGS0008 within 1 h, and no other radioactive metabolites were found

The metabolite profiling of MGS0274 was conducted with rat, monkey, and human cryopreserved hepatocytes and [^14^C]MGS0274 besylate. In all the species tested, a predominant peak was detected in RI‐HPLC analyses without other metabolite peaks after 1 hour incubation (Figure 2) and identified as MGS0008 based on the chromatographic retention time and mass spectral data (Table S3).

### 3.3 Hydrolytic activity against MGS0274 in rat, monkey, and human sera and tissue S9 fractions

The results are summarized in Table 1. In rats, all the tissue S9 fractions exhibited almost equal activity levels, followed by the serum. In monkeys, the hydrolytic activity was equally high in S9 fractions from the intestine, liver, and kidney, followed by the lung S9 fraction. In contrast to rats, monkey serum had no apparent hydrolytic activity against MGS0274. In humans, the liver S9 fraction exhibited the highest hydrolytic activity against MGS0274, followed by S9 fractions from the lung and intestine, while no apparent hydrolytic activity was observed in the serum or kidney S9 fraction.

**Table 1:** Hydrolytic activity against MGS0274 in sera and tissue S9 fractions of rats, monkeys, and humans

	Formation rate of MGS0008 (nmol/min/mg protein)				
	Serum	Intestine	Liver	Lung	Kidney
Rat	0.0146 (0.000365)	0.458 (0.00970)	0.928 (0.0625)	0.466 (0.0864)	0.859 (0.0122)
Monkey	0.0000563 (0.000000854)	2.10 (0.0763)	1.97 (0.0240)	0.349 (0.0152)	1.67 (0.0244)
Human	0.0000530 (0.000000344)	0.0453 (0.0109)	3.14 (0.0789)	0.369 (0.0682)	‐^a^

Values are presented as the mean of triplicate determinations, with SD in parentheses.

Formation rate was not calculated because MGS0008 was not detected in the reaction mixture.

### 3.4 Inhibition of cytochrome P450s

The inhibitory potential of MGS0008 (0.3‐100 µmol/L) and MGS0274 besylate (0.03‐10 µmol/L) on the specific activities of 7 CYP isoforms (CYP1A2, 2B6, 2C8, 2C9, 2C19, 2D6, and 3A) was evaluated in human liver microsomes. Both compounds had no inhibitory potential on any of the CYP isoforms over the concentration ranges tested (Table S4).

### 3.5 Induction of cytochrome P450s

The induction potential of MGS0008 (3‐30 µmol/L) and MGS0274 besylate (3‐30 µmol/L) on the mRNA expression of CYP1A2, 2B6, and 3A4 was evaluated in three lots of primary cultured cryopreserved human hepatocytes. Even at the maximum concentration, MGS0008 showed 0.9‐ to 1.1‐fold, 0.9‐ to 1.3‐fold, and 0.9‐ to 1.9‐fold changes, and MGS0274 besylate showed 1.1‐ to 1.4‐fold, 1.4‐ to 2.4‐fold, and 0.9‐ to 1.8‐fold changes in the mRNA expression levels of CYP1A2, 2B6, and 3A4, respectively. These results suggested that both compounds have no apparent induction potential on the CYP mRNA tested (Tables S5 and S6).

### 3.6 Pharmacokinetics of MGS0008 in rats

**Figure 3:**
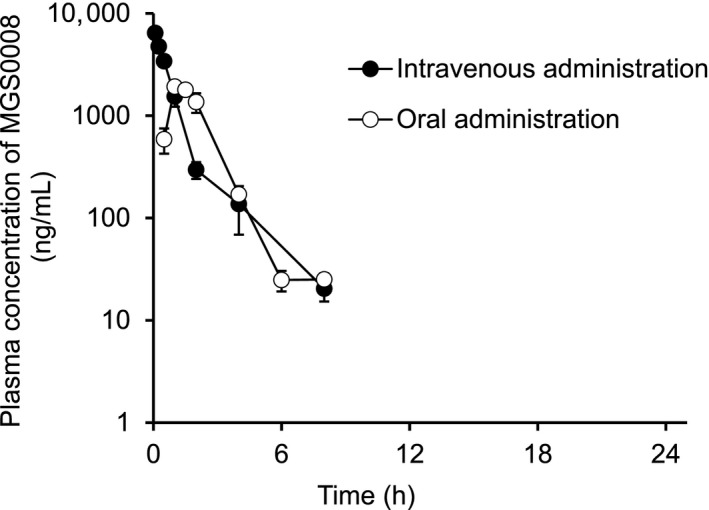
Plasma concentration‐time profiles of MGS0008 after a single intravenous or oral administration of MGS0008 to fasted male rats at a dose of 3 mg/kg. After intravenous administration, the MGS0008 level rapidly decreased, with a t_1/2_ of 1.54 h. After oral administration, the MGS0008 level reached a C_max_ of 1990 ng/mL and rapidly decreased, with a t_1/2_ of 0.968 h. The oral bioavailability was estimated to be 78.1%. Data are represented as the mean ± SD (n = 3). The plasma concentrations declined below the lower limit of quantification (3 ng/mL) at 24 h

The plasma concentration‐time profiles of MGS0008 after a single intravenous or oral administration of MGS0008 (3 mg/kg) to male rats under fasted conditions are shown in Figure 3, and the associated pharmacokinetic parameters are summarized in Table 2. After intravenous administration, the plasma MGS0008 level rapidly decreased, with a t_1/2_ of 1.54 hours, and the CL_total_ was calculated to be 548 mL/h/kg. The Vd_ss_ was estimated to be 545 mL/kg, indicating the extravascular distribution of MGS0008. In urine, 93.2% of the administered dose was excreted as the unchanged form within 48 hours, demonstrating metabolically stable properties in vivo and urinary clearance as the major route of elimination for MGS0008. The CL_renal_ was calculated to be 511 mL/h/kg from the amount of MGS0008 recovered in urine and the plasma AUC, which was approximately equal to the CL_total_ and glomerular filtration rate (GFR) in rats (516 ± 114 mL/h/kg),^9^ suggested that MGS0008 is less likely to undergo tubular secretion or reabsorption. After oral administration, MGS0008 reached a C_max_ of 1990 ng/mL at 1.17 hours postdose and rapidly declined, with a t_1/2_ of 0.968 hours. The oral bioavailability was estimated to be 78.1%.

**Table 2:** Pharmacokinetic parameters of MGS0008 after a single intravenous or oral administration of MGS0008 to fasted male rats at a dose of 3 mg/kg

Dosed compound		MGS0008	
Route		Intravenous	Oral
Dose		3 mg/kg	3 mg/kg
CL_total_	(mL/h/kg)	548 ± 29.4	–
Vd_ss_	(mL/kg)	545 ± 93.9	–
t_1/2_	(h)	1.54 ± 0.0600	0.968 ± 0.0310
C_max_	(ng/mL)	–	1990 ± 64.3
t_max_	(h)	–	1.17 ± 0.289
AUC_0–∞_	(h·ng/mL)	5480 ± 293	4280 ± 478
Bioavailability^a^	(%)	–	78.1
Urinary excretion	(%)	93.2 ± 4.69	NT
CL_renal_	(mL/h/kg)	511 ± 39.8	NT

Data are presented as the mean ± SD (n = 3) except for bioavailability which represents as the mean value of three animals.

–, not applicable; NT, not tested.

Bioavailability = (AUC_0‐∞_ following the oral administration) / (AUC_0‐∞_ following the intravenous administration) × 100

**Figure 4:**
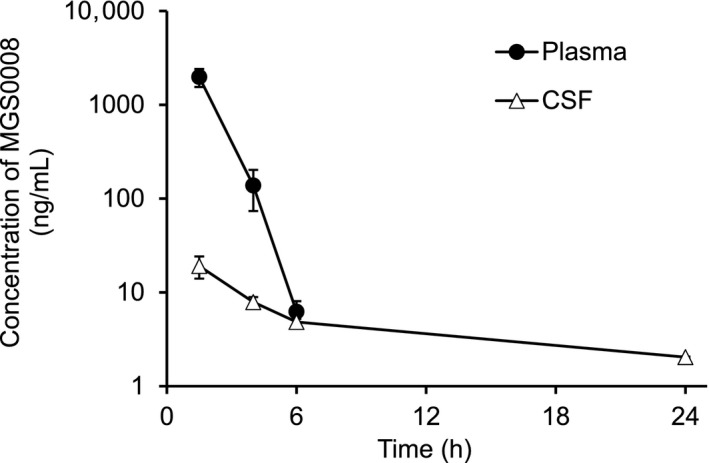
Plasma and cerebrospinal fluid (CSF) concentration‐time profiles of MGS0008 after a single oral administration of MGS0008 to fasted male rats at a dose of 3 mg/kg. At the first sampling time point (1.5 h), the concentration of MGS0008 in the CSF already reached a C_max_ value and then decreased at a slower rate than that observed in plasma. Data are represented as the mean ± SD (n = 3). The plasma concentration declined below the lower limit of quantification (3 ng/mL) at 24 h

The extent of CSF penetration of MGS0008 after a single oral administration of MGS0008 was also evaluated in fasted male rats at a dose of 3 mg/kg (Figure 4 and Table 3); at this dose, phencyclidine‐induced locomotor hyperactivity was reportedly inhibited. At the first sampling time point (1.5 hours), MGS0008 in the CSF had already reached a C_max_ value and then decreased at a slower rate than that observed in plasma. The AUC values in plasma and CSF were estimated to be 4330 h·ng/mL and 123 h·ng/mL, respectively. The AUC ratio of CSF to plasma was calculated to be 2.8%.

**Table 3:** Plasma and cerebrospinal fluid (CSF) concentrations of MGS0008, and pharmacokinetic parameters after a single oral administration of MGS0008 to fasted male rats at a dose of 3 mg/kg

Time	Plasma	CSF
(h)	(ng/mL)	(ng/mL)
1.5	1980 ± 431	19.1 ± 5.08
4	138 ± 64.2	7.88 ± 1.04
6	6.23 ± 1.83	4.84 ± 0.384
24	NC	2.04 ± 0.0265
AUC_0–24h_ (h·ng/mL)	4330 ± 719	123 ± 7.37
CSF/plasma AUC ratio (%)	–	2.8

Data are presented as the mean ± SD (n = 3).

The concentration in the plasma at 24 h postdose was less than the lower limit of quantification (3 ng/mL) and was treated as zero for calculation of AUC.

–, not applicable; NC, not calculated.

### 3.7 Pharmacokinetics of MGS0008 in monkeys

**Figure 5:**
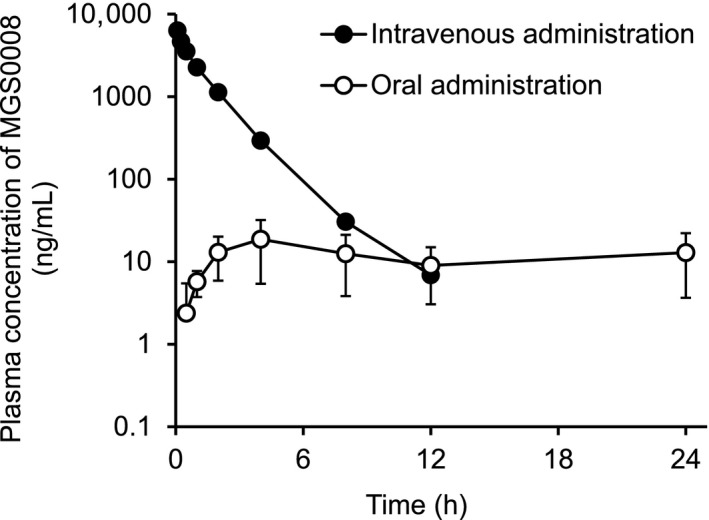
Plasma concentration‐time profiles of MGS0008 after a single intravenous or oral administration of MGS0008 to fed male monkeys at a dose of 1 mg/kg. After intravenous administration, the MGS0008 level rapidly decreased, with a t_1/2_ of 1.48 h, to be below the lower limit of quantification (3 ng/mL) at 24 h. After oral administration, the MGS0008 level reached a C_max_ of 20.5 ng/mL and showed sustained concentrations at low levels. The oral bioavailability was estimated to be 3.8%. Data are represented as the mean ± SD (n = 4)

The plasma concentration‐time profiles of MGS0008 after a single intravenous or oral administration of MGS0008 (1 mg/kg) to male monkeys under fed conditions are shown in Figure 5, and the associated pharmacokinetic parameters are summarized in Table 4. After intravenous administration, the plasma MGS0008 level rapidly decreased, with a t_1/2_ of 1.48 hours, and the CL_total_ was calculated to be 129 mL/h/kg. The Vd_ss_ was estimated to be 181 mL/kg, indicating the extravascular distribution of MGS0008. Similar to the observation in rats, 96.4% of the administered dose was excreted as the unchanged form in urine within 48 hours, and the CL_renal_ was calculated to be 124 mL/h/kg, which was approximately equal to the CL_total_ and GFR in monkeys (184 ± 30 mL/h/kg).^16^ Unlike the observations in rats, an oral dose of MGS0008 in monkeys resulted in sustained plasma concentrations at low levels during the observation period up to 24 hours, and the oral bioavailability of MGS0008 was calculated to be only 3.8%. These results, as well as the metabolically stable property of MGS0008, suggested a limited and gradual gastrointestinal absorption of MGS0008. Of note, three of the four monkeys experienced transient vomiting when MGS0008 was administered intravenously; however, the pharmacokinetic parameters were calculated for all the animals, since the vomiting was deemed not to have affected these values.

**Table 4:** Pharmacokinetic parameters of MGS0008 after a single intravenous or oral administration of MGS0008 to fed male monkeys at a dose of 1 mg/kg

Dosed compound		MGS0008	
Route		Intravenous	Oral
Dose		1 mg/kg	1 mg/kg
CL_total_	(mL/h/kg)	129 ± 13.3	–
Vd_ss_	(mL/kg)	181 ± 21.4	–
t_1/2_	(h)	1.48 ± 0.0435	NC
C_max_	(ng/mL)	–	20.5 ± 11.5
t_max_	(h)	–	9.00 ± 10.0
AUC_0–24h_^a^	(h·ng/mL)	7830 ± 776	281 ± 177
Bioavailability^b^	(%)	–	3.8 ± 2.8
Urinary excretion	(%)	96.4 ± 7.51	NT
CL_renal_	(mL/h/kg)	124 ± 19.7	NT

Data are presented as the mean ± SD (n = 4).

–, not applicable; NC, not calculated; NT, not tested.

Concentrations less than the lower limit of quantification (3 ng/mL) were treated as zero for calculation of AUC.

Bioavailability = (AUC_0‐24h_ following the oral administration) / (AUC_0‐24h_ following the intravenous administration) × 100

### 3.8 Pharmacokinetics of MGS0274 in monkeys

**Figure 6:**
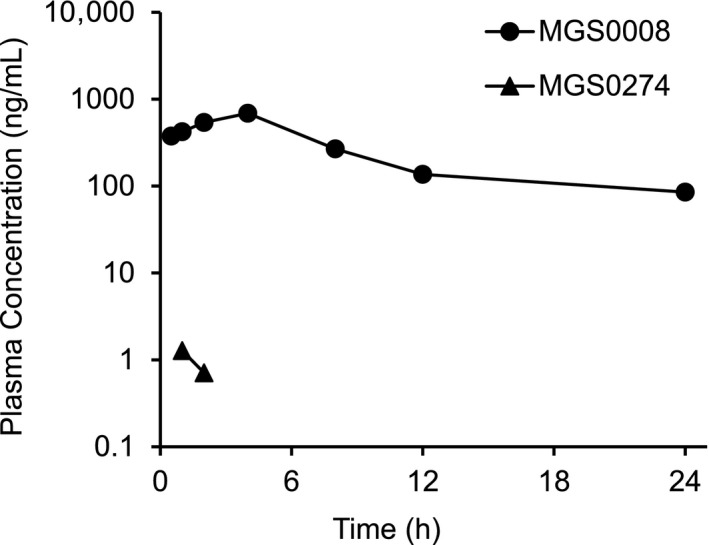
Plasma concentration‐time profiles of MGS0008 and MGS0274 after a single oral administration of MGS0274 besylate to fed male monkeys at a dose of 2.89 mg/kg (1‐mg equivalent of MGS0008/kg). After oral administration, MGS0274 was barely detectable at 1 and 2 h near the lower limit of quantification level (1 ng/mL). MGS0008 was detected from the first sampling time point (30 min), reached a C_max_ of 688 ng/mL at 4 h, and decreased, with a t_1/2_ of 16.7 h. The oral bioavailability of MGS0008 was estimate to be 83.7%. Data are represented as the mean of two animals

We administered MGS0274 besylate orally to fed male monkeys at a dose of 2.89 mg/kg (1‐mg equivalent of MGS0008/kg) and examined the plasma concentration profiles of MGS0274 and MGS0008 (Figure 6 and Table 5). Although MGS0274 was barely detected, MGS0008 was detected at the first sampling time point (30 minutes postdose) and reached a C_max_ of 688 ng/mL at 4 hours postdose, accounting for approximately 30‐fold over the C_max_ after the oral administration of MGS0008 (1 mg/kg). The oral bioavailability as MGS0008 was 83.7%, which was approximately 20‐fold higher than that after the oral administration of MGS0008 itself (3.8%). Moreover, the t_1/2_ of MGS0008 (16.7 hours) was apparently prolonged, compared with that observed after the intravenous administration of MGS0008 (1.48 hours). Of note, two of the four monkeys experienced vomiting at 12 and 19 minutes postdose in one animal and at 35 minutes and 4 hours postdose in the other animal after oral dosing of MGS0274 besylate. The pharmacokinetic parameters following the oral administration of MGS0274 besylate were calculated using data from the animals that did not vomit, since the vomiting may have affected these values.

**Table 5:** Pharmacokinetic parameters of MGS0008 and MGS0274 after a single oral administration of MGS0274 besylate to fed male monkeys at a dose of 2.89 mg/kg (1‐mg equivalent of MGS0008/kg)

Dosed compound		MGS0274 besylate	
Route		Oral	
Compound monitored		MGS0274	MGS0008
t_1/2_	(h)	NC	16.7
C_max_	(ng/mL)	1.28	688
t_max_	(h)	1.00	4.00
AUC_0–24h_^a^	(h·ng/mL)	2.03	6060
Bioavailability^b^	(%)	–	83.7

Data are presented as the mean of two animals.

–, not applicable; NC, not calculated.

Concentrations less than the lower limit of quantification (3 ng/mL) were treated as zero for calculation of AUC.

Bioavailability of MGS0008 =[AUC_0‐24h_ of MGS0008 following the oral administration of MGS0274 besylate (1‐mg equivalent of MGS0008/kg)] / [AUC_0‐24h_ of MGS0008 following the intravenous administration of MGS0008 (1 mg/kg)] × 100

### 3.9 Prediction of human pharmacokinetic parameters for MGS0008

The prediction of human pharmacokinetic parameters was performed using single‐species allometric scaling methods with the animal CL_total_, animal Vd_ss_, and unbound fraction. Since MGS0008 was unlikely to bind to plasma proteins in all the species tested, the unbound fraction value was set at 1. The CL_total_, Vd_ss_, and t_1/2_ in humans were predicted to be 146 mL/h/kg, 545 mL/kg, and 2.6 hours from the rat data, and 69.3 mL/h/kg, 181 mL/kg, and 1.8 hours from the monkey data, respectively.

## 4 DISCUSSION

MGS0008 is a rigid glutamate analog with a potent agonist activity for mGlu2/3 receptors and has been shown to exert antipsychotic actions in animal models of psychiatric disorders.^27^, ^36^ Based on the similarities in the chemical structures between MGS0008 and the preceding compounds, such as LY404039, the possibility that MGS0008 might exhibit poor oral bioavailability in humans was predicted. Furthermore, a certain level of a transportable prodrug of LY404039 (LY2140023) remained in plasma.^31^ Therefore, we decided to develop an ester‐based lipophilic prodrug to further reduce the systemic exposure to the prodrug itself. Currently, MGS0274 besylate, a prodrug of MGS0008, is being developed for the treatment of schizophrenia. Here, we investigated the disposition of MGS0274 and MGS0008 in rats and monkeys and their in vitro metabolism in humans to consider the developability of MGS0274 besylate as a prodrug.

First, the disposition of MGS0008 after intravenous administration in rats and monkeys was investigated, and we confirmed that almost all the administered compound was excreted as the unchanged form in urine. Moreover, no metabolites of MGS0008 were generated in the hepatocytes of rats and monkeys. These results indicate that the CL_total_ of MGS0008 is equivalent to the CL_renal_. The CL_renal_ values were almost equal to each GFR, suggesting that MGS0008 is less likely to undergo tubular secretion or reabsorption in the animal species that were tested. Although MGS0008 did not bind to plasma proteins in rats and monkeys, the Vd_ss_ were limited and roughly corresponded to the extracellular volumes. Thus, MGS0008 could conceivably penetrate into the extravascular spaces, but its hydrophilic property (cLogP = −1.18) makes it difficult to cross cell membranes easily, and this limitation is thought to influence its tissue distribution in rats and monkeys. Also, in human in vitro studies, MGS0008 was not metabolized in hepatocytes and did not bind to plasma proteins. Based on these results, similar distribution and excretion profiles of MGS0008 to those observed in the preclinical species seem possible in humans. In addition, a single‐species allometric scaling of the animal pharmacokinetic parameters demonstrated that the t_1/2_ of MGS0008 in human plasma is likely to be relatively short (1.8‐2.6 hours); this suggests that managing medication adherence could be difficult, since a reduced dosing frequency, such as once a day, is optimal, especially for the treatment of psychiatric disorders.^7^, ^25^ Of note, a single‐species allometric scaling method was used for the prediction of human pharmacokinetic parameters for the following reasons: it had reportedly performed better than in vitro‐in vivo extrapolation methods,^23^, ^33^ and the CL_total_ predicted from in vitro data had been underestimated in in‐house examination with a variety of compounds (data not shown).

In a previous study, the oral administration of MGS0008 to rats at a dose of 3 mg/kg exerted antipsychotic actions in an animal model of schizophrenia.^27^ Hence, the distribution of MGS0008 in the CSF as a surrogate for the central nervous system (CNS) exposure at this dose level in rats was evaluated. The AUC ratio of MGS0008 in the CSF relative to plasma was 2.8%, and MGS0008 exhibited a slower elimination from the CSF, compared with that from plasma. These characteristics, such as the limited penetration into the CNS and the slow elimination from the CNS, likely arise from the hydrophilic property of MGS0008. A similar pharmacokinetic profile was also reported in a study examining LY2812223 in humans, the chemical structure of which resembles that of MGS0008.^24^

The bioavailability after the oral administration of MGS0008 in monkeys was only 3.8%, whereas it was 78.1% in rats. Since MGS0008 was not metabolized in the preclinical species, the large species differences in oral bioavailability might be due to differences in the rates of its gastrointestinal absorption, such as transporter‐mediated uptake in rats. Species differences in oral bioavailability have also been reported for LY354740 (high in dogs and low in humans) 8, 17 and LY404039 (high in rats and low in humans).^2^, ^26^ MGS0008, which has a fluorine‐substituted structure of LY354740 at the C3 position, is also likely to exhibit low oral bioavailability in humans.

**Figure 7:**
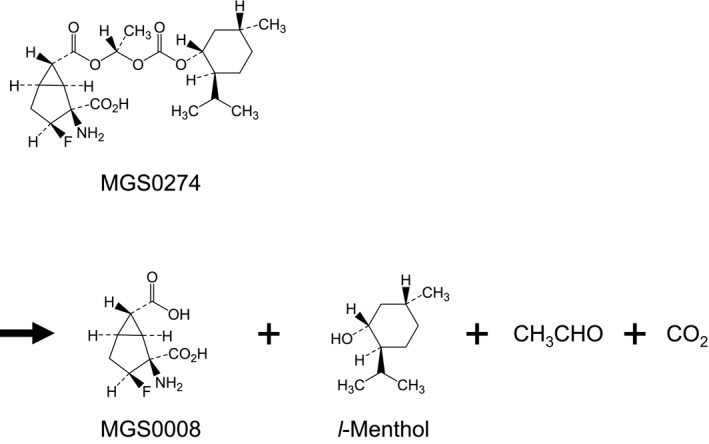
Hydrolytic conversion profile of MGS0274. MGS0274 is designed to liberate MGS0008, *l*‐menthol, acetaldehyde, and carbon dioxide, and this reaction is considered to be rapidly initiated by enzymatic hydrolysis and followed by spontaneous cleavage. This concept was supported by the in vitro metabolite profiling study as shown in Figure 2

MGS0274 is designed to liberate MGS0008, *l*‐menthol, acetaldehyde, and carbon dioxide (Figure 7), and this reaction is considered to be rapidly initiated by enzymatic hydrolysis and followed by spontaneous cleavage. Indeed, in vitro enzymatic hydrolysis of MGS0274 to MGS0008 was confirmed in all the species tested. As for *l*‐menthol and acetaldehyde, they were not directly detected in this study; however, there seems to be no remarkable species differences in their metabolism. In rats, monkeys, and humans, *l*‐menthol and acetaldehyde are thought to be metabolized to mainly *l*‐menthol glucuronide 10, 13, 40 and to acetate,^5^, ^20^, ^35^ respectively. Metabolites liberated from ester promoiety should be safe or low toxic. Indeed, acetaldehyde is a low toxic fragment which is rapidly detoxified, and there are many marketed prodrugs which release acetaldehyde via hydrolysis of their ester promoiety.^3^ On the other hand, *l*‐menthol has not been used as ester promoiety before. However, the systemic exposure level of *l*‐menthol after dosing of MGS0274 besylate is expected to be much lower than that after dosing of *l*‐menthol at 4 mg/kg, which is an acceptable daily intake 39 and equivalent to the amount of *l*‐menthol released from 15 mg/kg of MGS0274 besylate. Therefore, if a daily clinical dose of MGS0274 besylate is beyond this level, the systemic exposure to *l*‐menthol should be monitored and compared with those in animals from the perspectives of the Food and Drug Administration Guidance for Industry: Safety Testing of Drug Metabolites (2016, https://www.fda.gov/media/72279/download).

In this study, we confirmed that the hydrolytic activity against MGS0274 in the human liver S9 fraction was as high as that in the monkey liver S9 fraction and that MGS0274 was unstable in rat serum but stable in monkey and human sera. According to research on hydrolases, carboxylesterases (CESs), especially CES1, would be mainly responsible for the enzymatic hydrolysis of MGS0274 based on the structure of its ester promoiety.^15^ Indeed, rats have high levels of CES1 in serum, whereas humans and monkeys lack this hydrolase in sera.^34^, ^38^ Further studies will be conducted using recombinant enzymes to clarify which enzymes are responsible for the hydrolysis of MGS0274. Since the in vitro hydrolytic profile of MGS0274, the tissue distribution patterns of CES, and the potential risk of the low oral absorbability of MGS0008 in humans are deemed to resemble those in monkeys, the extrapolation of in vivo monkey pharmacokinetic data would be the most suitable approach to predicting in vivo human pharmacokinetics. To evaluate the oral absorbability of the prodrug, we dosed MGS0274 besylate orally to monkeys and confirmed an approximately 20‐fold improvement in the bioavailability of MGS0008 (from 3.8% to 83.7%). The result indicated that MGS0274 was stable in the gastrointestinal tract because MGS0008 had exhibited poor oral absorbability in monkeys. Note that the stability of MGS0274 in buffers, the pH of which were set at 1.2 and 6.5 as surrogate for gastrointestinal fluids, was confirmed (data not shown). Furthermore, MGS0274 was barely detected in monkey plasma, indicating the almost complete presystemic hydrolysis of MGS0274 to MGS0008 after gastrointestinal absorption. In addition, the plasma t_1/2_ of MGS0008 in monkeys (16.7 hours) was obviously prolonged, compared with that after the intravenous administration of MGS0008 (1.48 hours). The prolonged plasma t_1/2_ in monkeys is likely caused by the rate‐limiting release of MGS0008 into plasma from the enterocytes and/or hepatocytes, in which the hydrolysis of MGS0274 to MGS0008 occurs. Considering that MGS0008 in the CNS is eliminated more slowly than from plasma in rats, the same elimination profile in the CNS can be expected in monkeys and humans, allowing a reduced dosing frequency. Of note, MGS0274 besylate as well as MGS0008 did not have any apparent inhibition or induction potential on major CYP isoforms, indicating little likelihood of pharmacokinetic drug‐drug interactions via CYP in humans.

Consequently, we confirmed that MGS0274 was rapidly and extensively hydrolyzed to the parent compound MGS0008 after gastrointestinal absorption, and MGS0008 was distributed throughout the body without further metabolism and then excreted renally in the animal species that were tested. In monkeys, compared with MGS0008, an oral dose of MGS0274 besylate successfully improved the bioavailability of MGS0008 by up to approximately 20‐fold, with the prolonged plasma t_1/2_ of MGS0008. Furthermore, the hydrolytic activities against MGS0274 in the liver S9 fraction were comparable between monkeys and humans. Thus, MGS0274 besylate is expected to function properly as a prodrug and to exhibit a preferable pharmacokinetic profile for MGS0008 in humans, as it does in monkeys. Based on the preclinical data obtained, MGS0274 besylate has entered phase 1 clinical trials.

## DISCLOSURES

The authors declare that there is no conflict of interest.

## AUTHOR CONTRIBUTIONS

All authors are employees of Taisho Pharmaceutical Co., Ltd. Ochi, Fukasawa, Kinoshita, and Yamaguchi *participated in research design*. Ochi, Iwata, and Fukasawa *conducted experiments*. Ochi, Iwata, Fukasawa, Kinoshita, and Yamaguchi *performed data analysis*. Kinoshita, Ochi, Iwata, Fukasawa, and Yamaguchi* wrote or contributed to the writing of the manuscript*.
