# Supplementary material for: Preclinical disposition of MGS0274 besylate, a prodrug of a potent group II metabotropic glutamate receptor agonist MGS0008 for the treatment of schizophrenia
Source: Pharmacol Res Perspect. 2019 Sep 13;7(5):e00520. doi: 10.1002/prp2.520 (PMC6743422; doi:10.1002/prp2.520)
Supplement: Supplementary file 1 [file PRP2-7-e00520-s001.docx]

**Supplemental Table S1. Overview of experimental conditions for liquid chromatography-tandem mass spectrometry (LC-MS/MS), liquid chromatography-mass spectrometry (LC-MS), and high-performance liquid chromatography equipped with radiochemical flow detection (RI-HPLC).**

|  | Conditions |
| --- | --- |
| **A** | **For plasma protein binding study (LC-MS/MS)**  **[Column]** Shim-pack XR-ODS (2.2 μm, 30 mm × 3.0 mm I.D.; Shimadzu, Kyoto, Japan). Column temperature: 50°C.  **[Mobile phase]** (A) 0.1% v/v formic acid and (B) acetonitrile. Flow rate: 1.3 mL/min. Linear gradient: 0.00–1.00 min, from 20 to 98% B; 1.00–1.20 min, hold isocratic at 98% B; 1.20–1.21 min, from 98 to 20% B; 1.21–1.60 min, hold isocratic at 20% B.  **[Detection]** TripleQuad^TM^ 5500 (AB Sciex, Framingham, MA) with the TurboIonSpray ionization mode in the positive ion detection mode. MGS0274 (*m*/*z* 430→386) and [^2^H_4_]MGS0274 (*m*/*z* 434→390)*. |
| **B** | **For *in vitro* metabolite profiling for [^14^C]MGS0274 in hepatocytes (RI-HPLC and LC-MS)**  **[Column]** YMC-Pack ODS-AQ (3 μm, 150 mm × 4.6 mm I.D.; YMC, Kyoto, Japan). Column temperature: 40°C.  **[Mobile phase]** (A) 0.1% v/v acetic acid and (B) acetonitrile. Flow rate: 1.0 mL/min. Linear gradient: 0.00–10.00 min, hold isocratic at 1% B; 10.00–20.00 min, from 1 to 98% B; 20.00–25.00 min, hold isocratic at 98% B; 25.00–25.01 min, from 98 to 1% B; 25.01–30.00 min, hold isocratic at 1% B.  **[Detection]**  Radiomatic 625TR (PerkinElmer, Waltham, MA) with liquid scintillator (Flo-Scint^TM^ II, PerkinElmer) at 3 mL/min. LTQ Orbitrap XL (Thermo Fisher Scientific, Waltham, MA) with the heated electrospray ionization mode in the positive ion detection mode. |
| **C** | **For i*n vitro* metabolite profiling for MGS0274 in sera and tissue S9 fractions (LC-MS/MS)**  **[Column]** Xbridge Amide (3.5 μm, 50 mm × 4.6 mm I.D.; Waters, Milford, MA). Column temperature: 50°C.  **[Mobile phase]** (A) 0.1% v/v formic acid and (B) acetonitrile. Flow rate: 1.0 mL/min. Linear gradient: 0.00–0.30 min, hold isocratic at 90% B; 0.30–2.50 min, from 90 to 50% B; 2.50–3.00 min, hold isocratic 50% B; 3.00–3.01 min, from 50 to 90% B; 3.01–4.00 min, hold isocratic at 90% B.  **[Detection]** API4000^TM^ (AB Sciex) with the TurboIonSpray ionization mode in the positive ion detection mode. MGS0008 (*m*/*z* 204→158) and [^13^C_2_^15^N]MGS0008 (*m*/*z* 207→160)*. |
| **D** | **For inhibition study for cytochrome P450 (LC-MS/MS)**  **[Column]** Shim-pack XR-ODS (2.2 μm, 30 mm × 3.0 mm I.D.). Column temperature: 50°C.  **Method 1 for acetaminophen^a^, 4’-hydroxydiclofenac^b^, and 1’-hydroxybufuralol^a^**  **[Mobile phase]**  (A) 0.1% v/v formic acid and (B) acetonitrile. Flow rate: 1.5 mL/min. Linear gradient: 0.00–1.00 min, from 2 to 95% B; 1.00–1.20 min, hold isocratic 95% B; 1.20–1.21 min, from 95 to 2% B; 1.21–1.30 min, hold isocratic at 2% B.  **[Detection]** API4000^TM^ with the TurboIonSpray ionization mode in the positive ion detection mode. Acetaminophen (*m*/*z* 152→110), [^2^H_4_]*N*-(4-hydroxyphenyl)acetamide^c^ (*m*/*z* 156→114)*, 4’-hydroxydiclofenac (*m*/*z* 312→231), [^13^C_6_]4'-hydroxydiclofenac^d^ (*m*/*z* 318→237)*, 1’-hydroxybufuralol (*m*/*z* 278→186), and [^2^H_9_]1’-hydroxybufuralol^d^ (*m*/*z* 287→186)*.  **Method 2 for hydroxybupropion^b^, 4’-hydroxymephenytoin^a^, 1’-hydroxymidazolam^a^, and 6β-hydroxytestosterone^b^**  **[Mobile phase]** (A) 0.1% v/v formic acid and (B) acetonitrile. Flow rate: 1.5 mL/min. Linear gradient: 0.00–1.00 min, from 2 to 60% B; 1.00–1.01 min, from 60 to 95% B; 1.01–1.20 min, hold isocratic 95% B; 1.20–1.21 min, from 95 to 2% B; 1.21–1.30 min, hold isocratic at 2% B.  **[Detection]**  API4000^TM^ with the TurboIonSpray ionization mode in the positive ion detection mode. Hydroxybupropion (*m*/*z* 256→139), [^2^H_6_]hydroxybupropion^d^ (*m*/*z* 262→139)*, 4’-hydroxymephenytoin (*m*/*z* 235→150), [^2^H_3_]4'-hydroxymephenytoin^d^ (*m*/*z* 238→150)*, 1’-hydroxymidazolam (*m*/*z* 342→203), [^13^C_3_]1’-hydroxymidazolam^d^ (*m*/*z* 345→206)*, 6β-hydroxytestosterone (*m*/*z* 305→269), and [^2^H_7_]6β-hydroxytestosterone^d^ (*m*/*z* 312→276)*.  **Method 3 for *N*-desethylamodiaquine^b^**  **[Mobile phase]**  (A) 0.1% v/v formic acid and (B) acetonitrile. Flow rate: 1.3 mL/min. Linear gradient: 0.00–1.00 min, from 2 to 20% B; 1.00–1.01 min, from 20 to 95% B; 1.01–1.20 min, hold isocratic 95% B; 1.20–1.21 min, from 95 to 2% B; 1.21–1.50 min, hold isocratic at 2% B.  **[Detection]** TripleQuad5500^TM^ with the TurboIonSpray ionization mode in the positive ion detection mode. *N*-Desethylamodiaquine (*m*/*z* 328→283) and [^2^H_3_]*N*-desethylamodiaquine^d^ (*m*/*z* 331→283)*. |
| **E** | **For Pharmacokinetic analysis of MGS0008 (LC-MS/MS)**  **[Column]** XBridge Amide (3.5 μm, 50 mm × 4.6 mm I.D.). Column temperature: 50°C.  **[Mobile phase]** (A) 0.1% v/v formic acid and (B) acetonitrile. Flow rate: 1.0 mL/min. Linear gradient: [plasma and brain] 0.00–0.60 min, hold isocratic 90% B; 0.60–3.00 min, from 90 to 50% B; 3.00–3.50 min, hold isocratic at 50% B; 3.50–3.51 min, from 50 to 90% B; 3.51–4.50 min, hold isocratic at 90% B, or [CSF and urine] 0.00–0.60 min, hold isocratic 90% B; 0.60–4.00 min, from 90 to 65% B; 4.00–4.01 min, from 65 to 50% B; 4.01–4.50 min, hold isocratic at 50% B; 4.50–4.51 min, from 50 to 90% B; 4.51–5.50 min, hold isocratic at 90% B.  **[Detection]**  TripleQuad5500^TM^ with the TurboIonSpray ionization mode in the positive ion detection mode. MGS0008 (*m*/*z* 204→158) and [^13^C_2_^15^N]MGS0008 (*m*/*z* 207→160)*. |
| **F** | **For Pharmacokinetic analysis of MGS0274 (LC-MS/MS)**  **[Column]** Shim-pack XR-ODS (2.2 μm, 30 mm × 3.0 mm I.D.). Column temperature: 50°C.  **[Mobile phase]** (A) 0.1% v/v formic acid and (B) acetonitrile. Flow rate: 1.3 mL/min. Linear gradient: 0.00–1.00 min, from 2 to 98% B; 1.00–1.20 min, hold isocratic 98% B; 1.20–1.21 min, from 98 to 2% B; 1.21–1.70 min, hold isocratic at 2% B.  **[Detection]** TripleQuad5500^TM^ with the TurboIonSpray ionization mode in the positive ion detection mode. MGS0274 (*m*/*z* 430→386) and MGS0039 (*m*/*z* 378→159)*. |

*: internal standard

a: supplied by Sigma-Aldrich (currently Merck, St. Louis, MO)

b: supplied by BD Gentest (currently Corning, Corning, NY)

c: supplied by C/D/N Isotopes (Quebec, Canada)

d: supplied by BD Biosciences (San Jose, CA)

**Supplemental Table S2. Overview of experimental conditions for the *in vitro* studies on inhibitory effects of MGS0008 and MGS0274 besylate on the activity of human cytochrome P450 isoforms.**

| CYP isoform | Probe substrate | Probe reaction | Metabolite monitored | Reference inhibitor | Incubation period (min) |
| --- | --- | --- | --- | --- | --- |
| 1A2 | Phenacetin^a^  (40 μM) | Phenacetin  *O*-deethylation | Acetaminophen | Furafylline^b^  (1, 10 μM) | 10 |
| 2B6 | Bupropion^b^  (145 μM) | Bupropion  hydroxylation | Hydroxybupropion | Paroxetine^c^  (0.25, 2.5 μM) | 10 |
| 2C8 | Amodiaquine^d^  (1 μM) | Amodiaquine  *N*-deethylation | *N*-desethylamodiaquine | Montelukast^c^  (0.03, 0.3 μM) | 5 |
| 2C9 | Diclofenac^b^  (5 μM) | Diclofenac  4'-hydroxylation | 4'-Hydroxydiclofenac | Sulfaphenazole^b^  (0.1, 1 μM) | 5 |
| 2C19 | (*S*)-Mephenytoin^e^  (50 μM) | (*S*)-Mephenytoin  4'-hydroxylation | 4'-Hydroxymephenytoin | Tranylcypromine^b^  (1.5, 15 μM) | 10 |
| 2D6 | (±)-Bufuralol^e^  (10 μM) | Bufuralol  1'-hydroxylation | 1'-Hydroxybufuralol | Quinidine^b^  (0.02, 0.2 μM) | 10 |
| 3A | Midazolam^a^  (5 μM) | Midazolam  1'-hydroxylation | 1'-Hydroxymidazolam | Ketoconazole^a^  (0.01, 0.1 μM) | 5 |
| 3A | Testosterone^f^  (50 μM) | Testosterone  6β-hydroxylation | 6β-Hydroxytestosterone | Ketoconazole^a^  (0.01, 0.1 μM) | 10 |

The final protein concentration in the reaction mixture was set at 0.1 mg/mL. The values in parentheses represent the final concentrations of compounds added in each reaction mixture. Positive control inhibition was performed with reference inhibitors, of which the final concentrations in the reaction mixtures were selected to inhibit thoroughly each CYP isoforms (> 50% inhibition).

a: supplied by Wako Pure Chemical (currently FUJIFILM Wako Pure Chemical, Osaka, Japan)

b: supplied by Sigma-Aldrich (currently Merck, St. Louis, MO)

c: supplied by LKT Laboratories (St. Paul, MN)

d: supplied by MP Biomedicals (Santa Ana, CA)

e: supplied by Toronto Research Chemicals (Ontario, Canada)

f: supplied by Honeywell Fluka (Morris Plains, NJ)

Supplemental Table S3. LC-MS data and proposed product ions of MGS0274 and MGS0008 observed in the 1-hour incubation mixture of rat, monkey, and human cryopreserved hepatocytes with MGS0274 besylate.

| Metabolite | Retention time (min) | [M+H]^+^ | Characteristic product ions (*m/z*) | Description of the product ions | Samples |
| --- | --- | --- | --- | --- | --- |
| MGS0274 | 19.1 | 430 | 386  342  204 | loss of CH_3_CHO from the ion (*m*/*z* 430)  loss of CO_2_ from the ion (*m*/*z* 386)  MGS0008 (*m*/*z* 204) | R, M, H |
| MGS0008 | 4.9 | 204 | 186  158 | loss of H_2_O from the ion (*m*/*z* 204)  loss of CO from the ion (*m*/*z* 186) | R, M, H |

R: incubation mixture of rat hepatocytes with MGS0274 besylate

M: incubation mixture of monkey hepatocytes with MGS0274 besylate

H: incubation mixture of human hepatocytes with MGS0274 besylate

**Supplemental Table S4. IC_50_ values of MGS0008 and MGS0274 besylate for CYP inhibition in human liver microsomes.**

| CYP isoforms | Probe substrate | Probe metabolite | IC_50_ (μM)^a^ | |
| --- | --- | --- | --- | --- |
|  |  |  | MGS0008 | MGS0274 besylate |
| 1A2 | Phenacetin | Acetaminophen | > 100 | > 10 |
| 2B6 | Bupropion | Hydroxybupropion | > 100 | > 10 |
| 2C8 | Amodiaquine | *N*-desethylamodiaquine | > 100 | > 10 |
| 2C9 | Diclofenac | 4'-Hydroxydiclofenac | > 100 | > 10 |
| 2C19 | (*S*)-Mephenytoin | 4'-Hydroxymephenytoin | > 100 | > 10 |
| 2D6 | (±)-Bufuralol | 1'-Hydroxybufuralol | > 100 | > 10 |
| 3A | Midazolam | 1'-Hydroxymidazolam | > 100 | > 10 |
| 3A | Testosterone | 6β-Hydroxytestosterone | > 100 | > 10 |

a: The IC_50_ (half maximal inhibitory concentration) value was expressed as > 100 μM or > 10 μM because the percent inhibition in the presence of MGS0008 (100 μM) and MGS0274 besylate (10 μM), respectively, was less than 50% in all the CYP isoforms tested.

**Supplemental Table S5. Effect of MGS0008 on the CYP1A2, 2B6, and 3A4 mRNA expression levels in the primary cultured cryopreserved human hepatocytes.**

| Hepatocyte  Lot number | Compound | Concentration (μM) | Fold change of mRNA expression level | | |
| --- | --- | --- | --- | --- | --- |
|  |  |  | CYP1A2 | CYP2B6 | CYP3A4 |
| Hu8114 | MGS0008 | 3 | 1.1 | 0.8 | 1.4 |
|  |  | 10 | 1.0 | 0.8 | 1.4 |
|  |  | 30 | 1.1 | 1.0 | 1.9 |
|  | Positive control*^a^* | — | 20.7 | 35.1 | 44.6 |
| Hu8123 | MGS0008 | 3 | 0.9 | 1.2 | 1.0 |
|  |  | 10 | 1.1*^b^* | 1.2*^b^* | 0.7 |
|  |  | 30 | 0.9 | 1.3 | 0.9 |
|  | Positive control*^a^* | — | 59.2 | 45.7 | 23.0 |
| Hu1487 | MGS0008 | 3 | 0.8 | 0.9 | 1.3 |
|  |  | 10 | 0.7 | 0.7 | 1.3 |
|  |  | 30 | 1.0 | 0.9 | 1.7 |
|  | Positive control*^a^* | — | 35.9 | 16.4 | 19.8 |

Data are presented as the mean of triplicate determinations, except those marked as duplicate.

MGS0008 did not show detectable toxicity to the cultured human hepatocytes at the concentrations tested.

a: Positive controls are omeprazole^c^ (50 μM) for CYP1A2, phenobarbital^c^ (1000 μM) for CYP2B6, and rifampicin^d^ (10 μM) for CYP3A4.

b: duplicate determination.

c: supplied by Wako Pure Chemical (currently FUJIFILM Wako Pure Chemical, Osaka, Japan)

d: supplied by Sigma-Aldrich (currently Merck, St. Louis, MO)

**Supplemental Table S6. Effect of MGS0274 besylate on CYP1A2, 2B6, and 3A4 mRNA expression levels in the primary cultured cryopreserved human hepatocytes.**

| Hepatocyte  Lot number | Compound | Concentration (μM) | Fold change of mRNA expression level | | |
| --- | --- | --- | --- | --- | --- |
|  |  |  | CYP1A2 | CYP2B6 | CYP3A4 |
| Hu8114 | MGS0274 besylate | 3 | 1.1 | 0.8 | 1.1 |
|  |  | 10 | 1.0 | 1.0 | 0.9 |
|  |  | 30 | 1.1 | 1.5 | 1.1 |
|  | Positive control*^a^* | — | 20.7 | 35.1 | 44.6 |
| Hu8123 | MGS0274 besylate | 3 | 1.1 | 1.7 | 0.8 |
|  |  | 10 | 1.2 | 1.8 | 0.8 |
|  |  | 30 | 1.3 | 2.4 | 0.9 |
|  | Positive control*^a^* | — | 59.2 | 45.7 | 23.0 |
| Hu1487 | MGS0274 besylate | 3 | 1.0 | 1.1 | 1.4 |
|  |  | 10 | 1.0 | 1.0 | 1.5 |
|  |  | 30 | 1.4 | 1.4 | 1.8 |
|  | Positive control*^a^* | — | 35.9 | 16.4 | 19.8 |

Data are presented as the mean of triplicate determinations.

MGS0274 besylate did not show detectable toxicity to the cultured human hepatocytes at the concentrations tested.

a: Positive controls are omeprazole (50 μM) for CYP1A2, phenobarbital (1000 μM) for CYP2B6, and rifampicin (10 μM) for CYP3A4.
